# Longitudinal Between- and Within-Person Associations Among Screen Time, Bedtime, and Daytime Sleepiness Among Adolescents: Three-Wave Prospective Panel Study

**DOI:** 10.2196/78972

**Published:** 2026-01-21

**Authors:** Michał Tkaczyk, Albert J Ksinan, David Smahel

**Affiliations:** 1 Interdisciplinary Research Team on Internet and Society Faculty of Social Studies Masaryk University Brno Czech Republic; 2 RECETOX Faculty of Science Masaryk University Brno Czech Republic

**Keywords:** adolescents, bedtime, daytime sleepiness, random intercept cross-lagged panel model, RI-CLPM, screen restriction, screen time

## Abstract

**Background:**

Daytime sleepiness is prevalent among adolescents and linked to multiple health and functional impairments. Prior research has identified digital media use and insufficient sleep as key predictors, yet the reciprocal longitudinal associations among screen time, sleep, and daytime sleepiness remain understudied.

**Objective:**

This study examined the between- and within-person reciprocal longitudinal associations among adolescents’ screen time, bedtime, and daytime sleepiness. It also tested whether potential adverse effects of screen time were less pronounced over time among adolescents who limited their screen exposure before sleep at baseline.

**Methods:**

We conducted a prospective 3-wave panel study at 6-month intervals in a quota-based sample of 2500 Czech adolescents (mean age 13.43, SD 1.70 years; 1250/2500, 50% girls). Data were collected through an online survey. Screen time was assessed with 3 items covering total use of computers, smartphones, tablets, and television on a typical school day; bedtime with a single item on usual bedtime before school days; and daytime sleepiness with 4 items from the Pediatric Daytime Sleepiness Scale. Screen time restriction within 1 hour before sleep at baseline was examined as a moderator. Age and sex were included as covariates.

**Results:**

Between- and within-person associations were estimated using random intercept cross-lagged panel models. Adolescents with higher screen time reported later bedtimes (*r*=0.23, 95% CI 0.15-0.31; *P*<.001) and greater daytime sleepiness (*r*=0.25, 95% CI 0.16-0.34; *P*<.001). No direct within-person effects emerged between screen time and daytime sleepiness (W1→W2: β=.02, 95% CI –0.11 to 0.16; *P*=.71; W2→W3: β=.02, 95% CI –0.10 to 0.14; *P*=.66). However, increases in screen time at Wave 1 predicted later bedtime at Wave 2 (β=.14, 95% CI 0.01-0.27; *P*<.05), which in turn predicted higher screen time at Wave 3 (β=.24, 95% CI 0.11-0.36; *P*<.001). Temporary within-person spikes in screen time coincided with delayed bedtimes (W1: *r*=0.16, 95% CI 0.04-0.27; *P*<.01; W2: *r*=0.23, 95% CI 0.09-0.36; *P*<.001; W3: *r*=0.09, 95% CI 0.00-0.20; *P*=.049). Baseline screen time restriction did not moderate within-person effects (Δ*χ*^2^_6_=5.3; *P*=.51).

**Conclusions:**

This study is the first to test reciprocal longitudinal associations among adolescents’ screen time, bedtime, and daytime sleepiness while separating between- and within-person associations, thereby addressing potential bias common in prior cross-lagged panel studies. The findings refine theoretical understanding by indicating a complex, bidirectional, and mutually reinforcing interplay between screen time and bedtime over time—even when individual differences are accounted for—whereas daytime sleepiness does not appear to be affected by this dynamic. Additionally, negatively correlated, within-person fluctuations in screen time and bedtime indicate that these behaviors are partly mutually exclusive and likely shaped by shared contextual influences. Screen time restriction before sleep did not mitigate within-person effects, indicating that interventions should prioritize consistent sleep schedules rather than focusing solely on reducing screen use.

## Introduction

### Background

Daytime sleepiness is an important, yet understudied, dimension of adolescents’ sleep health [1]. Its prevalence varies widely across countries, ranging from 7.8% to 55.8% [2], and it is notably higher in adolescence than in adulthood [3]. Importantly, daytime sleepiness plays a central role in mediating the adverse effects of sleep impairment on adolescent health and well-being [4]. Studies have linked it to lower health-related quality of life [5], depressive symptoms, anxiety [6], heightened risk of mood disorders [7], and lower educational achievement [8]. Given its central role in linking sleep impairment to adverse outcomes, understanding the factors and processes contributing to daytime sleepiness in adolescence warrants greater scholarly attention.

Daytime sleepiness arises from an interplay of intrinsic (eg, brain maturation and sleep disorders) and extrinsic (eg, early school start times and poor sleep hygiene) factors [4]. Among these, insufficient sleep and late bedtimes on schooldays have been identified as the most direct contributors to daytime sleepiness among adolescents [9]. Digital media use is an important extrinsic factor known to affect sleep duration and bedtime timing; yet, most research on this association has been cross-sectional, limiting causal interpretations and leaving the direction of effects unclear [10,11].

Although the number of longitudinal studies is increasing [12], the vast majority do not distinguish between-person from within-person associations, which can lead to misleading conclusions about causal effects [13,14]. Studies that do separate these effects typically focus on short-term dynamics, such as day-to-day changes [15-18], often in small convenience samples, which limits their relevance for understanding longer-term processes.

To address these gaps in prior research, this study is the first to examine the longitudinal, reciprocal associations among screen time, bedtime, and daytime sleepiness, accounting for both stable between-person differences and within-person processes.

The study also tests whether restricting screen time before sleep moderates these associations. Clarifying whether daytime sleepiness emerges primarily from stable between-person differences, dynamic within-person processes, or both can advance theoretical understanding of how digital media use and adolescent sleep health influence each other. It may also help determine whether interventions should target stable behavioral patterns—such as sleep-related lifestyle habits, household routines, or family norms around screen use—or instead focus on longer-term individual trajectories—such as gradual increases in screen time or seasonal shifts in bedtime habits—or integrate both approaches.

### Prior Work

#### Associations Among Screentime, Bedtime, and Daytime Sleepiness

Cross-sectional research consistently demonstrates positive associations between various screen-based activities—such as television watching, internet use, video gaming, and phone use—and both delayed bedtimes and increased daytime sleepiness [10,11]. Whereas this evidence cannot be a basis for causal interpretations, it suggests that, for some adolescents, higher screen time, later bedtimes, and greater daytime sleepiness tend to co-occur. This pattern likely reflects stable between-person differences that may be linked to external factors such as individual traits (eg, social anxiety), lifestyle demands (eg, extracurricular commitments), and family environment characteristics (eg, parenting style and household rules) [19-22].

The recent synthesis of evidence suggests that the causal link between screen time and sleep health is bidirectional, involving 2 potential pathways [23]. The screen-time-affecting-sleep pathway posits that media use, in particular before or after bedtime, contributes to shorter sleep duration and poorer sleep quality. Four explanatory mechanisms have been proposed: melatonin suppression due to blue light exposure, psychological arousal, displacement of sleep time, and sleep interruptions [24-26]. Of these, only displacement—that is, delayed bedtime due to screen time—and nighttime interruptions from notifications appear to have a substantial impact on sleep [23].

Conversely, the impaired-sleep-affecting-screen-time pathway posits that changes in sleep can contribute to increased media use. Three mechanisms explain this effect. Circadian phase shifts in puberty result in extended evening free time for media use [27,28]. Adolescents may use digital media to cope with sleep difficulties [23,29]. Daytime sleepiness is associated with more sedentary behavior, including prolonged screen time [30].

Longitudinal evidence supporting the 2 pathways is mixed. Some adolescent studies support the screen-time-affecting-sleep pathway (eg, meta-analysis by Pagano et al [12]), others report reciprocal associations [31,32], and some find minimal or no effects [33-35]. Evidence for the sleep-impairment-affecting-screen-time pathway exists, but in young adult samples [36]. There are also some longitudinal studies that found little or no support for either pathway or only marginal effects [33-35]. Such mixed findings may partly stem from conflating between-person and within-person associations in prior longitudinal studies.

To date, only 2 longitudinal studies have investigated the within-person associations between electronic media use and sleep-related outcomes—one focusing on daytime sleepiness [34] and the other on bedtime [33]. The former did not find significant within-person associations between the frequency of social media use and daytime sleepiness in Dutch adolescents (aged 11-15 years), but did find between-person associations [34]. The latter, a 5-wave study of Finnish adolescents (aged 13-14 years at baseline), found no lagged effects and limited evidence of concurrent within-person associations—higher-than-usual social media use coincided with later-than-usual bedtime, but only in wave 1 [33]. These sparse findings suggest that the link between media use and sleepiness may arise from stable individual differences rather than changes over time.

#### A Moderating Role of Screen Time Restriction Before Sleep

Not all screen use is equally adverse for sleep health. In particular, evening screen time is considered detrimental to adolescent sleep [23], and restricting it is a common sleep hygiene recommendation [37]. Among adolescents, presleep screen restriction often results from parent-set technology rules, which cross-sectional studies have linked to less screen use, an earlier bedtime, and longer sleep duration [23]. While many adolescents do not follow their parents’ technology rules and recommendations [38], research synthesis suggests that interventions aimed at reducing prebedtime screen use lead to modest improvements in bedtime and sleep duration [39]. Although this evidence suggests a potentially protective effect of reducing evening screen use, evidence on whether presleep screen restrictions moderate the longitudinal relationship between adolescents’ screen time and sleep health is largely missing.

#### Covariates

Both screen use and sleep health vary by age and sex and therefore are important to consider when interpreting associations among adolescents’ screen time and sleep health. In particular, older adolescents sleep less, go to bed later, and spend more time on screens, and younger adolescents are more likely to limit evening screen use [40-42]. Findings on sex differences in sleep are mixed. Some studies report no substantial differences [40], while others show girls sleep more than boys [43], or the reverse [44]. Daytime sleepiness findings are also inconsistent [45]. Sex differences in screen time are clearer. Boys exceed screen time limits more often [46], and sleep-disrupting screen activities differ—girls’ sleep is more affected by social media, while boys’ is impacted by video games [42]. Together, these patterns indicate that age and sex are important individual factors in understanding variation in adolescents’ screen use and sleep health.

### This Study

Prior longitudinal studies have rarely distinguished between stable between-person differences and within-person fluctuations in digital media use and sleep, leaving uncertainty about whether observed associations reflect enduring individual characteristics or dynamic changes over time [19]. The few adolescent studies that applied this distinction produced inconclusive results, with limited evidence for lagged or concurrent within-person effects [33,34]. To address this gap, this study extends prior work by examining the reciprocal longitudinal associations among adolescents’ screen time, bedtime, and daytime sleepiness while separating between- and within-person processes. This allows us to clarify whether screen time and sleep co-vary because they influence each other over time or because of stable individual differences among adolescents. Furthermore, testing the moderating role of screen time restriction before sleep provides evidence on whether this common sleep hygiene recommendation mitigates longer-term effects of screen use on sleep health.

Specifically, we hypothesize that adolescents with higher overall screen time go to bed later and experience greater daytime sleepiness (Hypothesis 1); that increases in screen time are associated with a corresponding delay in bedtime and an increase in daytime sleepiness at the subsequent wave (Hypothesis 2), as well as within the same wave (Hypothesis 3); that delayed bedtime and increased daytime sleepiness are each associated with a subsequent increase in screen time (Hypothesis 4). The within-person effects expected in Hypotheses 2-4 reflect changes relative to a person’s typical patterns. Finally, we hypothesize that within-person associations are weaker among adolescents who restrict their screen use before sleep (Hypothesis 5).

## Methods

### Ethical Considerations

The study was approved by the Research Ethics Committee at Masaryk University (EKV-2018-068). Before participation, respondents were informed about the nature and purpose of the survey, their right to decline involvement, and their ability to skip any questions by selecting the “I prefer not to say” option available for all items. Informed consent was obtained from both adolescents and parents. Parents were instructed not to be present during the adolescent survey to protect privacy. Adolescents were asked to indicate if an adult had observed or intervened. Although most caregivers appeared to comply, this could not be independently verified. All data were fully deidentified prior to analysis, and no identifying information was collected or stored. No identification of individual participants in any images of the manuscript or supplementary material is possible.

Participants received reward points equivalent to approximately US $4, added to the panelist’s account and redeemable as cash or for charity donations.

### Study Design and Setting

A longitudinal observational design was used. This 3-wave prospective panel study was a part of a larger multifocal study examining various aspects of adolescents’ use of information and computer technologies and their impact on well-being. The first wave of data collection took place in June 2021, the second in November and December 2021, and the third in May and June 2022, with approximately 6 months between each wave. This study adhered to the STROBE (Strengthening the Reporting of Observational Studies in Epidemiology) reporting guidelines [47]; the completed STROBE checklist is provided in Multimedia Appendix 1 [48].

### Participants

This study was conducted on a sample of 2500 Czech adolescents aged 11-16 years (mean age 13.43, SD 1.70 years; 1250/2500, 50% girls). Data were collected in the Czech Republic by an external research agency that recruited participants from existing online panels using face-to-face interviews, computer-assisted telephone interviewing, and online methods. Eligible participants were Czech households with at least 1 adolescent aged 11-16 years and a caregiver, enabling data collection from adolescent-parent dyads within the same household. Quota sampling was used to ensure equal representation of gender, age, and their combination and to ensure that the sample reflected the distribution of Czech households with children based on households’ socioeconomic status (head of the household’s education level) and place of residence (Nomenclature of Territorial Units for Statistics, level 3, municipality size, European Commission, 2020). Out of 2500 participants initially recruited at Wave 1, a total of 1654 completed Wave 2, corresponding to an attrition rate of 33.8% (846/2500). At Wave 3, a total of 1102 participants remained in the study. The overall attrition rate from Wave 1 to Wave 3 was 44.1% (1102/2500), with an incremental attrition rate of 33.4% (552/1654) between Wave 2 and Wave 3.

### Measures

#### Screen Time

Screen time was assessed with 3 items, each starting with the question: “How much time (hours and minutes) do you spend doing the following activities during a typical school day?” The three items were: (1) “using a computer (PC or notebook),” (2) “using a cell phone or tablet,” and (3) “watching TV, including various videos on TV (eg, DVD, Netflix).” In response to these items, respondents picked hours and minutes using a time spinner. The screen time score was then computed by adding up the scores of each item.

#### Bedtime

Bedtime was measured with 1 item: “When do you usually go to bed before school days?” In response to this item, respondents picked hours and minutes using a time spinner.

#### Daytime Sleepiness

Daytime sleepiness was measured using 4 items from the Pediatric Daytime Sleepiness Scale, which contains 8 items assessing the frequency of specific daytime sleepiness symptoms [49]. The 4 items were “You get sleepy or drowsy while doing your homework,” “You have trouble getting out of bed in the morning,” “You tell yourself that you need more sleep,” and “You are tired and grumpy during the day.” The items were rated on a 5-point scale: (1) “never,”(2) “rarely,” (3) “sometimes,” (4) “often,” and (5) “very often.” For each measurement occasion, a composite score was calculated as the mean of the items measuring the construct. A higher score indicates higher daytime sleepiness. Cronbach α was computed to assess the reliability of the scale across 3 waves. Reliability estimates were: α=0.77 for Wave 1, α=0.81 for Wave 2, and α=0.82 for Wave 3. These results indicate that the scale has acceptable internal consistency over time. Mean scores of the observed items were used for daytime sleepiness in analyses due to convergence issues when the latent variable was incorporated into the trivariate random intercept cross-lagged panel model (RI-CLPM).

#### Screen Time Restriction Within 1 Hour Before Sleep

Screen time restriction within 1 hour before sleep was measured at Wave 1. First, respondents were asked: “How long before going to sleep do you usually stop using all devices with a screen, ie, phone, tablet, computer, television?” Respondents picked hours and minutes using a time spinner in response to this item. Then, these data were transformed into a binary variable with values of 0 for adolescents who reported less than 60 minutes and 1 for adolescents who reported 60 minutes or more.

#### Covariates

Sex and age at baseline were self-reported at Wave 1 and were both included as time-invariant covariates in the analysis. Sex was coded as 0 for girls and 1 for boys, and age was grouped into 11-13 years (0) and 14-16 years (1).

### Statistical Analysis

To examine the associations between screen time, bedtime, and daytime sleepiness over time while accounting for both between- and within-person sources of variance, we used RI-CLPMs fitted in *lavaan* (version 0.6-18) in R (version 4.4.1; R Core Team), allowing unbiased estimation of within-person effects net of stable individual differences [14]. The robust maximum likelihood estimator (MLR) was used, as it adjusts standard errors and chi-square statistics to accommodate nonnormal data (Section 3: “Testing normality assumptions” in supplementary materials provided by Tkaczyk et al [48]), yielding more accurate parameter estimates [50]. The proportion of missing data for the key time-varying variables ranged from 0.0% to 7.3% across waves. Little’s Missing Completely at Random (MCAR) test indicated that the data were not completely missing at random (*χ*²_377_=787.8; *P*<.001; normed *χ*²_377_=2.1), suggesting a small to moderate deviation from MCAR. Given the low proportion of missing data (<8% per variable), full information maximum likelihood (FIML) estimation was used to handle missing values. For a detailed breakdown of percentages of missingness for each variable and wave, and results of logistic regressions testing the relationship between key analytical variables, demographics, and dropouts are provided in Section 1: “Attrition analysis” in supplementary materials provided by Tkaczyk et al [48].

To obtain more robust estimates, nonparametric bootstrapping with 2000 resamples was used to estimate 95% CIs for both unstandardized and standardized effects. Standardized coefficients represent the SD change in outcomes per 1 SD change in exposure. Chi-square difference tests were used to compare the fit of a nested model with constraints to the fit of the unconstrained model unless otherwise specified. The modeling approach was adapted from Mulder and Hamaker [51]. In the first step, the unconstrained RI-CLPM was compared to a model where all random intercept variances and covariances were set to zero (statistically equivalent to cross-lagged panel model [CLPM]) to test for stable between-unit differences, using the chi-bar-square test [52]. The comparison showed that the RI-CLPM fit the data better (Δ*χ*^2^_6_=286.8; *P*<.001). In addition, random intercepts of all 3 constructs had significant variance, indicating that there were some stable between-person differences in screen time, bedtime, and daytime sleepiness over time. Second, to assess population-level changes in observed variables, we fixed grand means over time and compared this model to the unconstrained version. The comparison showed that the model without the constraints fit data better (Δ*χ*^2^_6_=74.1; *P*<.001), which implies that, on average, there was some change over time in all 3 variables. Third, to test whether the associations between screen time, bedtime, and daytime sleepiness were time-invariant, we constrained the autoregressive and cross-lagged paths, as well as the residual covariances. The model-building procedure indicated the fully unconstrained model as the best-fitting model Table 1. At this point, covariates (age and sex) were added to the model. The final model showed an adequate fit (*χ*^2^_15_=46.7; *P*<.001; Comparative Fit Index [CFI]=0.994; Tucker-Lewis Index [TLI]=0.977; root-mean-square error of approximation [RMSEA]=0.029, 90% CI 0.020-0.039; standardized root-mean-square residual [SRMR]=0.020). Fourth, moderation by screen time restriction before bed was tested using a multiple-group extension to RI-CLPM [51].

**Table 1:** Model fit indices for random intercept cross-lagged panel models (RI-CLPMs) examining longitudinal associations between screen time, bedtime, and daytime sleepiness across 3 waves in a longitudinal study of adolescents (aged 11-16 years). Data were collected in the Czech Republic between June 2021 and June 2022.

Model	*χ*²(*df*)	CFI^a^	SRMR^b^	RMSEA^c^	TLI^d^	AIC^e^	BIC^f^
M0^g^	7.3 (3)	0.999	0.010	0.024	0.989	45591.676	45888.702
M1^h^	294.0 (9)	0.938	0.044	0.113	0.750	45866.448	46128.530
M2^i^	81.4 (9)	0.984	0.026	0.057	0.937	45653.804	45915.886
M3^j^	42.7 (18)	0.995	0.023	0.029	0.989	45597.131	45806.796
M3 + Covs^k^	46.7 (15)	0.994	0.020	0.029	0.977	45301.270	45633.241

^a^CFI: Comparative Fit Index.

^b^SRMR: standardized root-mean-square residual.

^c^RMSEA: root-mean-square error of approximation.

^d^TLI: Tucker-Lewis Index.

^e^AIC: Akaike information criterion.

^f^BIC: Bayesian information criterion.

^g^M0: fully unconstrained RI-CLPM.

^h^M1: cross-lagged panel model [CLPM].

^i^M2: RI-CLPM with grand means constrained over time.

^j^M3: RI-CLPM with constraint over time imposed on auto-regressive paths, cross-lagged paths, and residual (co)variances.

^k^M3 + Covs: M0 with covariates (age and sex).

## Results

### Descriptive Analysis

Table 2 displays pairwise correlations for time-varying variables across waves, along with their descriptive statistics, skewness, and kurtosis. The means of daytime sleepiness are close to “sometimes” (Wave 1: 2.81, SD 0.80; Wave 2: 2.82, SD 0.82; Wave 3: 2.84, SD 0.82). At Wave 1, approximately every third (788/2500, 32%) participant reported having trouble getting out of bed in the morning often or very often. Getting sleepy or drowsy while doing homework was the least frequent symptom—at Wave 1, approximately every sixth (400/2494, 16%) participant reported experiencing it often or very often.

**Table 2:** Pearson correlations and descriptive statistics for screen time, bedtime, and daytime sleepiness across 3 waves in a longitudinal study of adolescents (aged 11-16 years). Data were collected in the Czech Republic between June 2021 and June 2022. All correlation coefficients (r) are significant at *P*<.001.

Variable	ST^a^ (W1^b^)	ST (W2^c^)	ST (W3^d^)	BT^e^ (W1)	BT (W2)	BT (W3)	DS^f^ (W1)	DS (W2)	DS (W3)
ST (W1), *r*	1.00								
ST (W2), *r*	0.63	1.00							
ST (W3), *r*	0.59	0.62	1.00						
BT (W1), *r*	0.23	0.16	0.14	1.00					
BT (W2), *r*	0.17	0.20	0.21	0.61	1.00				
BT (W3), *r*	0.13	0.11	0.18	0.56	0.63	1.00			
DS (W1), *r*	0.13	0.09	0.09	0.22	0.17	0.13	1.00		
DS (W2), *r*	0.15	0.15	0.13	0.20	0.23	0.18	0.57	1.00	
DS (W3), *r*	0.14	0.13	0.16	0.21	0.19	0.20	0.55	0.64	1.00
Mean (SD), hh:mm or scale	06:23 (02:40)	06:11 (02:36)	06:02 (02:37)	09:48 00:56	09:47 (00:56)	09:57 (00:58)	2.81 (0.80)	2.82 (0.82)	2.84 (0.82)
Skewness	0.38	0.51	0.59	0.04	0.31	0.26	0.15	0.13	0.10
Kurtosis	−0.54	−0.29	−0.23	0.28	0.83	0.58	0.14	0.11	0.09

^a^ST: screen time (hh:mm).

^b^W1: Wave 1.

^c^W2: Wave 2.

^d^W3: Wave 3.

^e^BT: bedtime (hh:mm PM).

^f^DS: daytime sleepiness (1-5).

Average bedtimes at each wave were before 10 PM (Wave 1: 9:48, SD 00:56; Wave 2: 9:47, SD 00:56; Wave 3: 9:57, SD 00:58). At Wave 1, a total of 14% (353/2465) of participants reported bedtime at 11:00 PM or later (213/1621, 13% at Wave 2 and 190/1093, 17% at Wave 3). Average total daily screen times were close to 6 hours at each wave and showed a decreasing tendency across time (Wave 1: 06:23, SD 02:40; Wave 2: 06:11, SD 02:36; Wave 3: 06:02, SD 02:37).

Intraclass correlation coefficients (ICCs) revealed that between-person differences accounted for approximately 64% of the variance in screen time, 60% in bedtime, and 58% in daytime sleepiness, indicating a smaller but substantial proportion of variance due to within-person changes over time. All variables showed statistically significant and positive correlations both within and across waves.

### Between-Person Associations Among Screen Time, Bedtime, and Daytime Sleepiness

**Figure 1:**
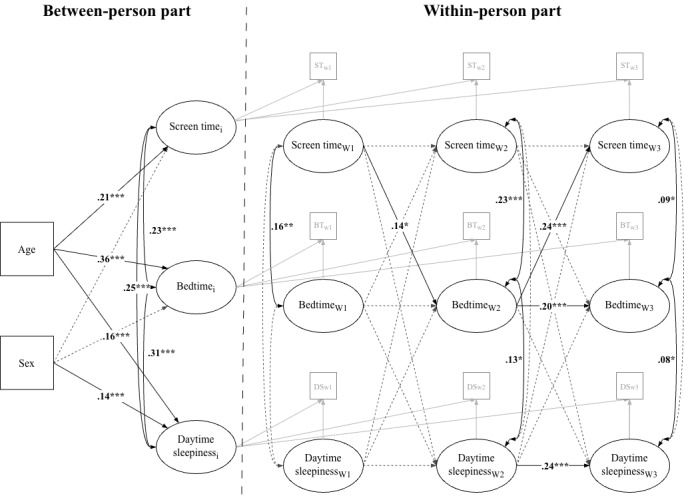
Standardized path coefficients of the final random intercept cross-lagged panel model testing between- and within-person associations among screen time, bedtime, and daily sleepiness across 3 measurement waves in a longitudinal study of adolescents (aged 11-16 years) conducted in the Czech Republic between June 2021 and June 2022. The model controls for the effects of age (at Wave 1 [W1]) and sex on the random intercepts of the time-varying variables. Solid black lines represent significant paths, and dashed lines represent nonsignificant paths. Solid gray paths were fixed to 1. *P<.05; **P<.01; ***P<.001.

Standardized path coefficients of the final RI-CLPM are presented in Figure 1.

The analysis revealed significant positive associations between the random intercepts of screen time and bedtime (*r*=0.23, 95% CI 0.15-0.31; *P*<.001), screen time and daytime sleepiness (*r*=0.25, 95% CI 0.16-0.34; *P*<.001), and bedtime and daytime sleepiness (*r*=0.31, 95% CI 0.22-0.41; *P*<.001). Consistent with Hypothesis 1, these correlations indicate that adolescents who typically use screens more also tend to go to bed later and experience higher daytime sleepiness. Additionally, those with later bedtimes tend to experience higher daytime sleepiness.

### Within-Person Associations Among Screen Time, Bedtime, and Daytime Sleepiness

The analysis identified 2 significant cross-lagged effects. Consistent with Hypothesis 2, elevated screen time at Wave 1 relative to a person’s usual patterns, was associated with elevated bedtime at Wave 2 (β=.14, 95% CI 0.01-0.27; *P*=.02). Similarly, in line with Hypothesis 4, elevated bedtime at Wave 2, relative to a person’s usual patterns, was associated with increased screen time at Wave 3 (β=.24, 95% CI 0.11-0.36; *P*<.001). No evidence was found for the remaining cross-lagged paths hypothesized in Hypothesis 2 or Hypothesis 4.

Consistent with Hypothesis 3, the analysis revealed consistent concurrent associations between the within-person components of screen time and bedtime (Wave 1: β=.16, 95% CI 0.04-0.27; *P*=.007; Wave 2: β=.23, 95% CI 0.010-0.36; *P*<.001; Wave 3: β=.09, 95% CI 0.01-0.19; *P*=.049), indicating that an increase in screen time—relative to a person’s usual patterns—was associated with a corresponding delay in bedtime with the same wave. No evidence was found to support Hypothesis 3. Additionally, a significant concurrent association between bedtime and daytime sleepiness was observed at Wave 2 (β=.13, 95% CI 0.01-0.26; *P*=.045) and Wave 3 (β=.08, 95% CI 0.00-0.17; *P*=.04). This indicates that, within these waves, a delay in bedtime was associated with elevated daytime sleepiness relative to a person’s usual level of sleepiness.

The analysis also revealed autoregressive effects. Elevated bedtime at Wave 2, relative to a person’s usual patterns, was associated with elevated bedtime at Wave 3 (β=.20, 95% CI 0.05-0.36; *P*<.001), indicating that a delay in bedtime—relative to a person’s usual patterns—tends to carry over time. A similar autoregressive effect was observed for daytime sleepiness, with elevated sleepiness at Wave 2 associated with elevated sleepiness at Wave 3 (β=.24, 95% CI 0.12-0.37; *P*<.001).

### The Role of Covariates

Age significantly predicted the intercepts of screen time (β=.21, 95% CI 0.16-0.25; *P*<.001), bedtime (β=.36, 95% CI 0.32-0.41; *P*<.001), and daytime sleepiness (β=.16, 95% CI 0.11-0.21; *P*<.001), indicating that older adolescents (aged 14-16 years) typically spent more time using screen media, have later bedtimes, and experience higher daytime sleepiness compared with younger adolescents (aged 11-13 years). Sex (boy=1) significantly predicted the intercept of daytime sleepiness (β=.14, 95% CI 0.10-0.19; *P*<.001), indicating that typical levels of daytime sleepiness are higher for boys than for girls (Table 3).

**Table 3:** Estimated parameters of the random intercept cross-lagged panel model (RI-CLPM) testing between- and within-person associations of screen time, bedtime, and daytime sleepiness across 3 measurement waves in a longitudinal study of adolescents (aged 11-16 years) conducted in the Czech Republic between June 2021 and June 2022. The model controls for the effects of age (at Wave 1) and sex on the random intercepts of the time-varying variables.

Parameter			*B*		SE			95% CI		*P* value	β	
**Between-person associations**												
	**Correlations**											
		ST_i_^a^ ↔ BT_i_^b^		0.314		0.062	0.184 to 0.436		<.001			.229
		ST_i_ ↔ DS_i_^c^		0.296		0.050	0.177 to 0.407		<.001			.250
		BT_i_ ↔ DS_i_		0.123		0.019	0.084 to 0.159		<.001			.312
	**Covariates**											
		Age → ST_i_		0.859		0.100	0.658 to 1.05		<.001			.206
		Sex → ST_i_		0.050		0.099	−0.138 to 0.247		.62			.012
		Age → BT_i_		0.523		0.033	0.457 to 0.591		<.001			.362
		Sex → BT_i_		0.054		0.033	−0.012 to 0.112		.08			.038
		Age → DS_i_		0.191		0.029	0.135 to 0.248		<.001			.160
		Sex → DS_i_		0.172		0.028	0.113 to 0.228		<.001			.144
**Within-person associations**												
	**Autoregressive paths**											
		ST^d^ (W1^e^) → ST (W2^f^)		0.100		0.070	−0.049 to 0.235		.15			.100
		BT^g^ (W1) → BT (W2)		0.047		0.081	−0.145 to 0.233		.56			.045
		DS^h^ (W1) → DS (W2)		0.027		0.074	−0.147 to 0.183		.72			.025
		ST (W2) → ST (W3^i^)		0.056		0.068	−0.103 to 0.201		.41			.056
		BT (W2) → BT (W3)		0.224		0.059	0.04 to 0.387		<.001			.200
		DS (W2) → DS (W3)		0.245		0.051	0.112 to 0.362		<.001			.242
	**Cross-lagged paths**											
		ST (W1) → BT (W2)		0.051		0.023	0.002 to 0.099		.03			.139
		ST (W1) → DS (W2)		0.008		0.020	−0.037 to 0.051		.71			.023
		BT (W1) → ST (W2)		0.131		0.171	−0.238 to 0.463		.44			.046
		BT (W1) → DS (W2)		0.006		0.057	−0.124 to 0.133		.92			.006
		DS (W1) → ST (W2)		0.001		0.186	−0.411 to 0.398		>.99			.000
		DS (W1) → BT (W2)		-0.018		0.072	−0.178 to 0.125		.81			−.015
		ST (W2) → BT (W3)		-0.009		0.022	−0.057 to 0.037		.69			−.021
		ST (W2) → DS (W3)		0.008		0.018	−0.037 to 0.051		.66			.024
		BT (W2) → ST (W3)		0.635		0.162	0.309 to 0.976		<.001			.235
		BT (W2) → DS (W3)		-0.010		0.044	−0.113 to 0.084		.83			−.011
		DS (W2) → ST (W3)		0.116		0.173	−0.25 to 0.481		.50			.038
		DS (W2) → BT (W3)		0.102		0.061	−0.025 to 0.23		.09			.080
	**Residual covariances**											
		ST (W1) ↔ BT (W1)		0.156		0.058	0.04 to 0.277		.007			.158
		ST (W1)↔ DS (W1)		0.043		0.047	−0.059 to 0.156		.05			.050
		BT (W1) ↔ DS (W1)		0.025		0.018	−0.011 to 0.063		.15			.084
		ST (W2) ↔ BT (W2)		0.229		0.066	0.087 to 0.364		<.001			.225
		ST (W2) ↔ DS (W2)		0.098		0.057	−0.021 to 0.213		.09			.108
		BT (W2) ↔ DS (W2)		0.042		0.021	−0.005 to 0.088		.045			.127
		ST (W3) ↔ BT (W3)		0.099		0.050	−0.008 to 0.209		.049			.091
		ST (W3) ↔ DS (W3)		0.077		0.040	−0.006 to 0.159		.06			.090
		BT (W3) ↔ DS (W3)		0.030		0.014	−0.001 to 0.061		.04			.083

^a^ST_i_: screen time latent intercept.

^b^BT_i_: bedtime latent intercept.

^c^DS_i_: daytime sleepiness latent intercept.

^d^ST: screen time.

^e^W1: Wave 1.

^f^W2: Wave 2.

^g^BT: bedtime.

^h^DS: daytime sleepiness.

^i^W3: Wave 3.

### The Moderating Role of Screen Time Restriction Before Bed

Against Hypothesis 5, comparisons of multiple group RI-CLPMs with and without constraints across groups showed no differences in correlations between random intercepts (Δ*χ*^2^_3_=6.0; *P*=.11), residual covariances (Δ*χ*^2^_6_=3.5; *P*=.74), or cross-lagged associations (Δ*χ*^2^_6_=5.3; *P*=.51) across adolescents who restricted screen time 1 hour before bed at Wave 1 and those who did not. However, some significant differences between those groups were found (Δ*χ*^2^_2_=32.0; *P*<.001). Adolescents who restricted their screen time before bed reported, on average, shorter screen time (by 27 minutes and 28 seconds), earlier bedtime (22 minutes and 12 seconds), and lower daytime sleepiness (Δ=0.159; Table 3).

## Discussion

### Principal Results

This 3-wave prospective panel study examined bidirectional relationships between screen time, bedtime, and daytime sleepiness in a large representative sample of early to midadolescents in the Czech Republic. Findings at the between-person level showed that higher screen time, later bedtimes, and increased daytime sleepiness tend to co-occur among adolescents. At the within-person level, results revealed a bidirectional, transactional association between screen time and bedtime, suggesting mutual reinforcement over time. Additionally, temporary, wave-specific deviations in screen time and bedtime—relative to a person’s usual patterns—were positively correlated, suggesting that increases in screen time and delays in bedtime tend to co-occur within individuals at the same wave. Finally, while restricting screen time before sleep did not modify these associations, adolescents who restricted screen time had lower typical screen time, earlier bedtimes, and less daytime sleepiness on average.

### Between-Person Associations Among Screen Time, Bedtime, and Daytime Sleepiness

Consistent with Hypothesis 1, the analysis revealed small to medium positive correlations between screen time, bedtime, and daytime sleepiness at the between-person level, aligning with findings from cross-sectional studies [4,10,11,53]. However, previous RI-CLPM studies reported mixed correlation patterns. For instance, Maksniemi et al [33] found no significant between-person correlations between active social media use and bedtime, whereas 2 other studies reported medium positive correlations between social media use and daytime sleepiness [34] and between media multitasking and sleep problems [35]. Such inconsistencies may reflect differences across studies in how media use was conceptualized and defined (eg, active vs general social media use).

The between-person associations observed in this study indicate that higher screen time and poorer sleep co‐occur as relatively stable individual tendencies, likely shaped by other stable factors. For example, late chronotype may predispose some adolescents to later bedtimes and heavier evening media use [54]. Prior work has shown that modifiable factors—such as parenting style [55], parental sleep [21], media habits [56], and household rules [57,58]—also influence both adolescent media habits and sleep. To guide better-targeted interventions, future longitudinal RI-CLPM studies should investigate how various modifiable family and lifestyle factors influence the media–sleep association over time.

### Within-Person Associations Among Screen Time, Bedtime, and Daytime Sleepiness Over Time

Consistent with Hypothesis 2, increased screen time was associated with delayed bedtime 6 months later, but only between Waves 1 and 2. According to the interpretation guidelines proposed by Orth et al [59], the effect is considered large. The association, although not consistent across all waves, aligns with prior longitudinal research, including a 6-wave study based on data from the ABCD study among adolescents aged 11‑14 years [60] and a 2-wave study among adolescents aged 13-14 years [31], both of which link media use to later bedtimes over time. The present findings extend the prior evidence by demonstrating the association even when controlling for stable between‐person differences. Other RI-CLPM studies did not find cross-lagged effects; for instance, Maksniemi et al [33] found no association between active social media use and bedtime. Such discrepancies may reflect differences in conceptualizing media use—overall screen time versus active social media use—which involve distinct pathways linking media to sleep. Whereas active social media use mainly disrupts sleep through presleep arousal [61], total screen time is more closely related to blue light exposure and sleep displacement, the latter showing stronger and substantial associations with reduced sleep duration [23].

Contrary to Hypothesis 2, this study found no evidence of a direct within-person association between screen time and daytime sleepiness in the long term. This result is consistent with 2 previous RI-CLPM studies. Van Der Schuur et al [34] also found no evidence of a long-term within-person association between social media use and daytime sleepiness, aside from a small effect of social media stress among girls. Van der Schuur et al [35] found no direct path from media multitasking to sleep problems (including daytime sleepiness), except for a marginally significant effect of media multitasking among girls. Although direct effects were absent, indirect pathways remain plausible. Daytime sleepiness may occur when screen use results in later bedtimes [24]. Although bedtime was not formally tested as a mediator in this study, which should be considered a limitation, future longitudinal studies might examine bedtime delay as a pathway linking screen time to daytime sleepiness.

Consistent with Hypothesis 3, temporary increases in screen time coincided with temporary delays in bedtime across all 3 waves, indicating concurrent within-person associations between the two. Similar results were found by Maksniemi et al [33] in a single wave, whereas other RI-CLPM studies did not examine concurrent associations [34,35]. This pattern likely reflects the mutually exclusive nature of screen use and sleep within daily time allocation [62]; yet, the association manifests itself in period-specific, typical patterns of behavior—during periods when bedtime is delayed, adolescents have more opportunities for screen use, and conversely, during periods with greater screen use, they have less time available for sleep. Findings further indicate that bedtime remains sensitive to short-term, period-specific changes in screen time (and vice versa) and that both may share common contextual drivers.

Against Hypothesis 3, this study found no evidence of a correlated change between screen time and daytime sleepiness, suggesting that short-term increases in screen time do not directly coincide with increased sleepiness. Similarly, a diary study on smartphone use and next-day sleepiness found no such effects [18]. Delayed bedtimes in Waves 2 and 3 were concurrently linked to increased daytime sleepiness, likely due to shorter sleep duration [63]. Overall, the pattern of longitudinal associations found in this study suggests that while screen time and daytime sleepiness are not directly linked at the within-person level, an indirect path is possible, whereby delayed bedtime may mediate the association between technology use and daytime sleepiness.

Consistent with Hypothesis 4, this study found a within-person cross-lagged effect of bedtime on screen time in the subsequent wave: a later-than-usual bedtime predicted increased screen time 6 months later, but only between Waves 2 and 3. This finding aligns with prior longitudinal research showing reciprocal effects between poorer sleep and greater media use [31,36]. The RI-CLPM study by Van der Schuur et al [34] provided partial evidence for the opposite direction, with increased daytime sleepiness predicting decreased social media use over time among boys. Unlike earlier RI-CLPM studies, this study supports the sleep-impairment-affecting-screen-time pathway, demonstrating a substantial effect even after accounting for stable between-person differences. Although this effect was not consistent across all waves, it suggests that adolescents may extend screen use to fill additional evening hours, which likely arises from circadian shifts or related factors [28].

Overall, discrepancies in cross-lagged effects across RI-CLPM studies may partly reflect differences in the time intervals between measurements. This study used a 6-month interval, whereas Van der Schuur et al [34,35] used a 3- to 4-month interval, and Maksniemi et al [33] used a 1-year interval. The absence of cross-lagged effects in some cases suggests that these intervals may not have been optimal for capturing the underlying dynamics [33]. Future research could benefit from greater use of different temporal designs, such as shortitudinals, to identify optimal temporal windows for detecting within-person effects and the temporal dynamics through which media use influences sleep across adolescence.

Taken together, the cross-lagged pattern (screen time → bedtime between Waves 1 and 2; bedtime → screen time between Waves 2 and 3) suggests a reinforcing cycle between increased screen time and delayed bedtime over time. While previous research identified bidirectional links between screen time and sleep [31], this study extends prior work by being the first to demonstrate this reinforcing pattern longitudinally using an RI-CLPM that accounts for stable between-person differences. The autoregressive effects further indicate that delayed bedtimes tend to carry over across waves—a finding also reported in other RI-CLPM studies [33], which may reflect adolescent circadian shifts or habitual delays associated with greater autonomy or increased school demands [9]. Considering that delayed bedtimes were concurrently linked to greater daytime sleepiness and prospectively to higher screen time, interventions that promote earlier and more consistent sleep schedules, rather than solely limiting screen use, may be more effective for improving adolescent sleep health.

### Effects of Screen Time Restriction Before Sleep

Contrary to Hypothesis 5, this study found no evidence that restricting screen time before sleep affected within-person associations between screen time and sleep, particularly regarding the development of sleep displacement over time. Prior findings are mixed—while experimental studies have shown improvements in sleep outcomes [64,65], observational studies often report no adverse effects of prebedtime smartphone use [17,18], with inconsistent adherence to parent-set rules frequently cited as a limiting factor [61]. These discrepancies likely reflect differences in study design, sampling strategies, and time frames (eg, short- vs long-term). It should also be noted that the comparison groups were defined based on screen time restriction assessed at Wave 1 only. However, this behavior was not stable over time—among those who reported limiting their screen use at Wave 1, only 40% (284/710) did so across all 3 waves, and 65% (460/710) did so at least once thereafter. Future research should account for this temporal variability when examining the long-term effects of screen time restriction.

Adolescents who reported restricting screen use before sleep also tended to report lower overall screen exposure, earlier bedtimes, and less daytime sleepiness than their peers. Although these between-person differences may indicate a protective role of screen time restriction, they could also reflect other stable characteristics such as family environment (eg, parenting style), chronotype, or self-regulation. Prior research has linked adverse parenting styles to poorer sleep quality and greater daytime sleepiness [55], and greater sleepiness to lower self-regulation and eveningness chronotype [66]. Future longitudinal studies should account for these factors and examine their potential moderating roles in the relationship between screen use and sleep outcomes.

### Limitations

Several limitations should be considered when interpreting these findings. First, the study relied on self-reported measures of screen time and sleep, which may be prone to inaccuracy [67,68]. Because overall screen time was calculated by summing reported use across multiple devices that could have been used simultaneously, average values may overestimate actual exposure. Future research should integrate digital trace data [69] and wrist-worn accelerometers data [70] for more accurate measurements.

Second, measurement simplifications—using total screen time and an abbreviated version of the Pediatric Daytime Sleepiness Scale [49]—may have reduced precision and obscured associations with sleep [71]. Future studies should use more detailed measures that account for media functions, content, and context of use [72,73].

Third, with only three waves spaced 6 months apart, the design was insufficient for modeling longer-term developmental trajectories [53,74] or accounting for seasonal variability in screen time and sleep [75,76]. Longer follow-up and more frequent measurement occasions would allow finer modeling of these changes.

Fourth, attrition was higher than in comparable school-based studies [33-35], likely because data were collected through an online panel and required the agreement of both adolescent and parent or caregiver. Online panels typically exhibit higher attrition rates due to the sustained participant burden and email-based recontact [77,78], and similar rates have been reported in other adolescent panel studies [79]. Notably, attrition remained high despite offering substantially increased incentives (160% in Waves 1 and 2; 280% in Wave 3). Dropouts reported slightly higher baseline screen time (Tables S1 and S2 in supplementary materials provided by Tkaczyk et al [48]), which may limit generalizability to heavy screen users.

Finally, data collection partially overlapped with COVID-19 social distancing measures, which were associated with increased screen time and later bedtimes among adolescents [60,80]. The stringency of restrictions varied across waves: Wave 1 (June 2021) coincided with the strictest measures, Wave 2 (November-December 2021) with moderate restrictions, and Wave 3 (May-June 2022) after their removal [81]. This variation may partly explain the observed decrease in screen time and the stability of bedtime between Waves 1 and 2.

### Conclusion

This study is the first to test reciprocal longitudinal associations among adolescents’ screen time, bedtime, and daytime sleepiness while separating between- and within-person processes, thereby addressing bias common in prior cross-lagged panel studies. The findings refine theoretical understanding by showing a complex, bidirectional, and mutually reinforcing interplay between screen time and bedtime over time, even after accounting for stable individual differences. Between-person associations revealed that adolescents with higher screen use had poorer sleep, likely reflecting the influence of relatively stable individual and environmental factors. Although specific cross-lagged effects varied across waves, the overall pattern supports both the screen-time-affecting-sleep and sleep-impairment-affecting-screen-time pathways, whereas daytime sleepiness was not affected by this dynamic. Negatively correlated within-person fluctuations further indicate that screen time and bedtime are partly mutually exclusive and may share contextual drivers.

Screen time restriction before sleep did not moderate within-person effects. However, at the between-person level, adolescents who practiced it reported lower screen use, earlier bedtimes, and less daytime sleepiness. Taken together, these findings suggest that interventions emphasizing consistent sleep schedules and supportive family routines—rather than focusing solely on limiting screen use—may be most effective for promoting adolescent sleep health. Future research should incorporate objective measurements on multiple time scales and relevant moderators.

## Appendix

Multimedia Appendix 1 STROBE checklist.

## Abbreviations

CFI: Comparative Fit Index
CLPM: cross-lagged panel model
FIML: full information maximum likelihood
ICC: intraclass correlation coefficient
MCAR: Missing Completely at Random
MLR: maximum likelihood estimator
RI-CLPM: random intercept cross-lagged panel model
RMSEA: root-mean-square error of approximation
SRMR: standardized root-mean-square residual
STROBE: Strengthening the Reporting of Observational Studies in Epidemiology
TLI: Tucker-Lewis Index

## Data Availability

The data used in this study are openly available on the Open Science Framework (OSF) [48].
